# Large diameter scleral lens benefits for Asians with intractable ocular surface diseases: a prospective, single-arm clinical trial

**DOI:** 10.1038/s41598-021-82010-z

**Published:** 2021-01-27

**Authors:** Jayoon Moon, Sang-Mok Lee, Joon Young Hyon, Mee Kum Kim, Joo Youn Oh, Hyuk Jin Choi

**Affiliations:** 1 Department of Ophthalmology, Seoul National University College of Medicine, Seoul, Republic of Korea; 2 Laboratory of Ocular Regenerative Medicine and Immunology, Seoul Artificial Eye Center, Seoul National University Hospital Biomedical Research Institute, Seoul, Republic of Korea; 3 Department of Cornea, External Disease & Refractive Surgery, HanGil Eye Hospital, Incheon, Republic of Korea; 4 Department of Ophthalmology, Catholic Kwandong University College of Medicine, Gangneung-si, Gangwon-do Republic of Korea; 5 Department of Ophthalmology, Seoul National University Bundang Hospital, Seongnam-si, Gyeonggi-do Republic of Korea; 6 Department of Ophthalmology, Seoul National University Hospital Healthcare System Gangnam Center, 39Th Fl., Gangnam Finance Center, 152 Teheran-ro, Gangnam-gu, Seoul, 06236 Republic of Korea

**Keywords:** Clinical trial design, Corneal diseases

## Abstract

To report the efficacy and safety of large diameter scleral lenses and determine their suitability in Asian subjects with intractable ocular surface diseases. This prospective study enrolled intractable ocular surface diseases subjects with uncorrected visual acuity > counting finger but ≥ 0.3 logMAR and best-corrected visual acuity (BCVA) ≥ 0.3 logMAR, to fit large diameter scleral lenses for 12 weeks. 21 eyes (13 subjects) consisting ten eyes (47.6%) with persistent epithelial defects, 6 (28.6%) with graft-versus-host disease, 4 (19.0%) with Stevens–Johnson syndrome and one (4.8%) with severe dry eye were ultimately enrolled. Primary outcome measures were the visual acuity, corneal and conjunctival fluorescein staining, Ocular Surface Disease Index (OSDI), and National Eye Institute 25-Item Visual Function Questionnaire (NEI-VFQ-25). At week 12 with large diameter scleral lenses, BCVA improved from 0.77 logMAR to 0.27 logMAR (*P* < 0.001). High-grade corneal and conjunctival fluorescein staining proportion decreased from 61.90 to 14.29% and 52.38 to 9.52%, respectively (*P* = 0.0036 and 0.0063, respectively). OSDI and NEI-VFQ-25 improved from 67.89 to 34.69 and 51.40 to 64.48, respectively (*P* < 0.001). No adverse effects were observed. In Asians with intractable ocular surface diseases, large diameter scleral lens improves visual acuity and alleviates signs and symptoms of ocular surface diseases without any significant complications.

*Trial registration* Korean Health Technology R&D Project, Ministry of Health & Welfare, Republic of Korea (Project No. HI12C0015 (A120018)). Clinical Trials.gov, NCT04535388. Registered 18 August 2020—Retrospectively registered, http://clinicaltrials.gov/ct2/show/NCT04535388.

## Introduction

Scleral lenses are large-diameter rigid gas-permeable (RGP) contact lenses that are mostly supported by the sclera. Since the first introduction of gas-permeable scleral lenses in 1983, scleral lenses have been widely used for various ocular surface diseases^1,2^. By neutralizing optical aberrations, scleral lens can provide significant improvement in visual acuity for various irregular corneal conditions, such as severe keratoconus and postpenetrating keratoplasty^1–4^. Scleral lens can adequately retain tear reservoir between the posterior surface of the scleral lens and the cornea to efficaciously manage severe dry eye in a variety of chronic ocular surface diseases^1,5–9^.

Intractable ocular surface diseases are a collection of conditions with sustained ocular pain, permanent visual decrement due to persistent ocular inflammation, keratinization or conjunctivalization of the cornea and limbal stem cell deficiencies. Despite the development of new medications, supportive topical eyedrops still have limited therapeutic effects that insufficiently meet patient needs^10,11^, while surgical interventions including corneal or limbal transplantation are invasive procedures that can often result in failure due to rejection^12^. Therefore, a therapeutic approach such as scleral lens that is less invasive and has adequate clinical benefits in managing and treating intractable ocular surface diseases is necessary^1,13^. Over the years, scleral lens has emerged as an efficacious treatment option for various types of intractable ocular surface diseases with many advantageous results^1,2,6,9,14^. Scleral lens can successively enhance visual acuity in intractable ocular surface diseases when glasses are ineffective^1–4^. Also, scleral lens can significantly alleviate subjective dry eye symptoms^1–4^.

There are several commercially manufactured scleral lenses with different designs and sizes, all of which have been proven to be clinically efficacious in a wide spectrum of diseases^6^. Particularly in the management of intractable ocular surface diseases, scleral lenses with large overall diameters (OADs) are frequently used in order for the lens to entirely rest on the sclera and contain sufficient tear reservoir^1,13,15,16^. The prosthetic replacement of the ocular surface ecosystem (PROSE) devices (Boston Foundation for Sight, Needham, MA), one of the first and representative scleral lenses renowned for long-term clinical benefits in several complex corneal diseases, have large OADs ranging from 17.5 to 23.0 mm^17^. On the other hand, in the Republic of Korea, only scleral lenses with small OADs are currently available. SoClear and Onefit scleral lenses, two of the most commonly used scleral lenses in the Republic of Korea, possess OADs ranging from 14.1 to 15.5 mm and 13.3 to 15.0 mm, respectively, and are closer to corneoscleral type lenses. Compared to the Western population, Asians have smaller palpebral fissures with tighter eyelids which may restrict patients’ tolerability of wearing a large scleral lens and consequently limit its clinical benefits. Hence a more suitable scleral lens for Asians with adequately larger OAD for maximal clinical benefits is required.

The LK scleral lens (Lucid Korea LTD, Seoul, Republic of Korea) is made from a gas-permeable fluorosilicone acrylate polymer, Boston XO (hexafocon A; Polymer Technology, a Bausch & Lomb company, Wilmington, MA, USA) and has a Dk value of 100 × 10^–11^ cm^2^ · mL O2/sec · mL · mmHg. The OAD ranges from 13.0 to 24.0 mm, which is larger than other commercially available scleral lenses commonly used in the Republic of Korea.

Accordingly, we report the efficacy and safety of the LK scleral lens, which has been newly manufactured to possess larger OADs than currently available commercial lenses, and determine its suitability in Asian subjects with intractable ocular surface diseases.

## Results

### General demographics and medical history

A total of 16 subjects (total 25 eyes) were initially enrolled; 3 subjects were excluded for the following reasons: 2 subjects (3 eyes) met the exclusion criteria, and 1 subject (1 eye) failed to wear the LK scleral lens properly. Ultimately, 21 eyes of 13 subjects (age 20–59 years, average 38.5 years) were studied. Table 1 shows the general demographics and ocular conditions of the subjects at the screening visit who eventually finished the clinical trial. The studied ocular surface diseases were composed of the following: 10 eyes (47.6%) with persistent epithelial defect (PED) from various causes, 6 eyes (28.6%) with chronic graft-versus-host disease (cGVHD), 4 eyes (19.0%) with Stevens–Johnson syndrome (SJS) and 1 eye (4.8%) with severe dry eye syndrome.

**Table 1:** General demographics of patients ultimately enrolled at screening.

Patient	Age (years)	Sex	Laterality	Diagnosis	UCVA (logMAR)	BCVA (logMAR)	Oxford scale	OSDI	NEI-VFQ-25	CCT (μm)	ECD (cells/mm^2^)
1	23	M	R	SJS	0.82	0.82	4	95.8	73	336	*hazy*
			L		0.52	0.52	2			466	3003
2	43	M	R	SJS	1.40	1.40	4	*NA*	*NA*	293	hazy
			L		0.82	0.82	3			359	3105
3	20	M	R	PED^**a**^	0.52	0.52	3	38.6	65	379	3472
			L		1.40	1.40	3			271	*hazy*
4	46	F	L	PED^**b**^	0.30	0.30	4	56.3	78	*fail*	*hazy*
5	22	F	R	PED^**a**^	1.70	1.22	2	20.5	63.4	*fail*	*hazy*
			L		1.40	1.22	1			*fail*	*hazy*
6	58	M	L	PED^**c**^	0.82	0.82	4	61.1	36.5	629	*hazy*
7	37	F	R	PED^**d**^	0.82	0.70	2	85.4	70	618	2881
8	43	M	R	PED^**e**^	0.70	0.52	3	*NA*	*NA*	283	*hazy*
9	20	M	R	PED^**f**^	1.70	1.70	2	*NA*	*NA*	279	*hazy*
			L		2.00	2.00	2			*fail*	*hazy*
10	59	F	R	cGVHD	0.40	0.30	3	79.5	67	381	2857
			L		0.40	0.30	4			543	2617
11	53	F	R	cGVHD	0.30	0.30	3	100	70	266	3278
			L		0.40	0.40	3			259	2824
12	38	M	R	cGVHD	1.70	0.30	2	79.2	72	583	2857
			L		1.70	0.30	2			566	2941
13	39	F	L	DES	0.82	0.82	4	56.3	57.4	266	*hazy*
Mean ± SD	38.5 ± 13.9				0.98 ± 0.56	0.79 ± 0.51	2.9 ± 0.9	37.3 ± 25.4	65.2 ± 11.6	398.6 ± 138.0	2983.5 ± 246.0

^a^Aniridia associated limbal stem cell deficiency, ^b^Exposure keratopathy with neurotrophic keratitis, ^c^Exposure keratopathy, ^d^Exposure keratopathy associated with incomplete eyelid closure after upper eyelid blepharoplasty, ^e^Limbal stem cell deficiency after conjunctival squamous cell carcinoma in situ excisional operation with cryotherapy and topical interferon α2b application, ^f^Near total limbal stem cell deficiency due to contact lenses.

M: male, F: female, R: right, L: left, UCVA: uncorrected visual acuity, logMAR: log of minimal angle of resolution, BCVA: best-corrected visual acuity, SJS: Stevens–Johnsons Syndrome, PED: persistent epithelial defect, cGVHD: chronic graft-versus-host-disease, OSDI: ocular surface disease index, NEI-VFQ-25: National Eye Institute 25-Item Visual Function Questionnaire.

### Objective efficacy outcomes

**Figure 1:**
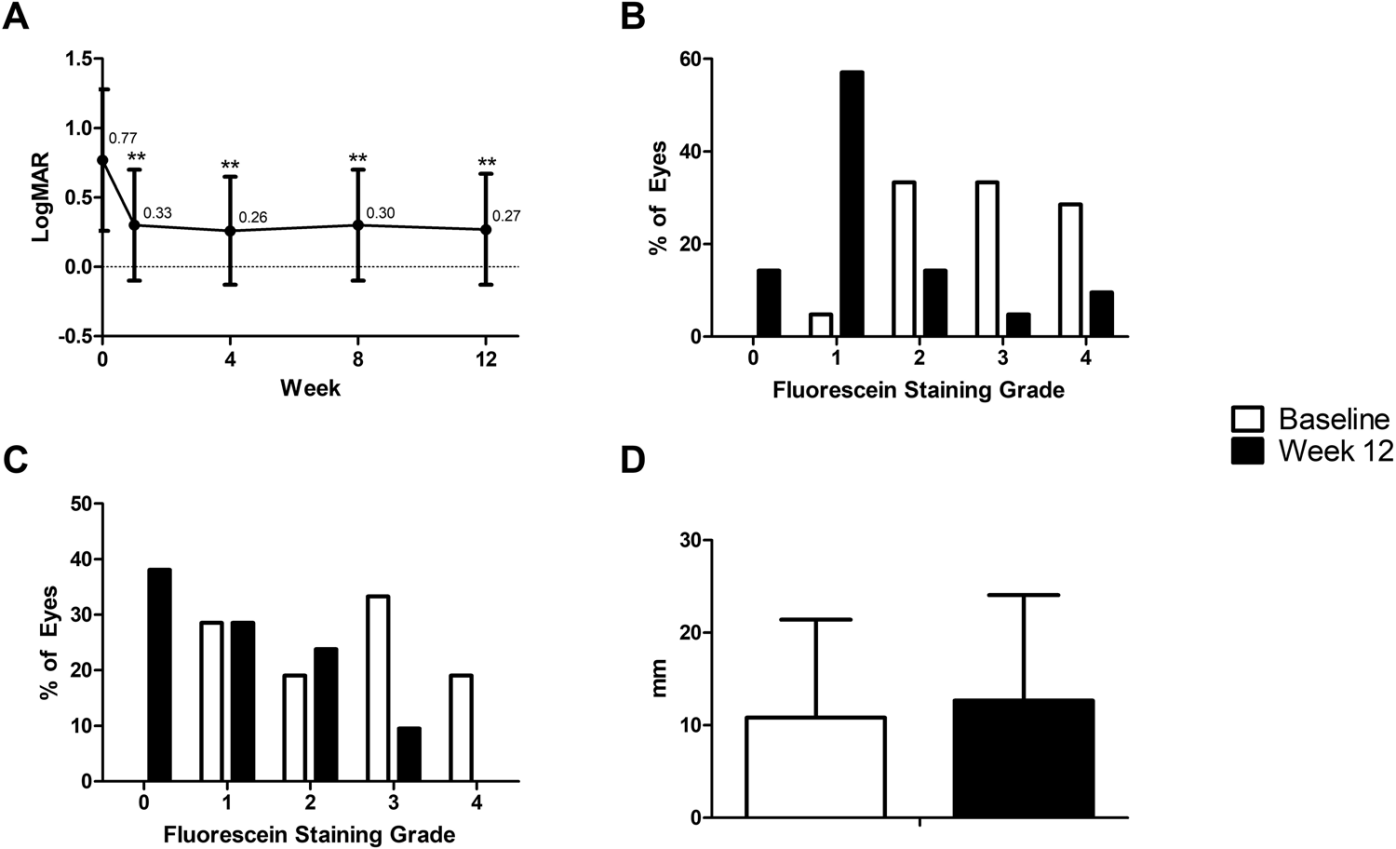
Objective efficacy outcomes of the LK scleral lens clinical trial. Significant improvement in BCVA was seen after 1 week of the LK scleral lens wearing and was stable throughout the 12-week study period. Baseline BCVA was 0.77 ± 0.51 logMAR (range 0.30–2.00) and improved to 0.30 ± 0.40 logMAR (range − 0.18–1.00), 0.26 ± 0.39 logMAR (range − 0.18–1.00), 0.30 ± 0.40 logMAR (range − 0.08–1.00) and 0.27 ± 0.40 logMAR (range − 0.08–1.00) at 1, 4, 8 and 12 weeks, respectively (compared to baseline; *P* < 0.001 at all weeks, paired t-test) (**A**). Baseline corneal fluorescein staining grade 3 or 4 was seen in 61.90% (13 eyes). After 12 weeks of LK scleral lens wearing, the proportion of corneal fluorescein staining grade 3 or 4 had decreased to 14.29% (3 eyes). A majority of subjects (71.43%, 15 eyes) were either grade 1 or 0 at week 12 (Grade 0–2 vs 3–4, from baseline to week 12; *P* = 0.0036, Fisher’s exact test) (**B**). Conjunctival fluorescein staining grade 3 or 4 were observed in 52.38% (11 eyes) and grades 1 or 0 in 28.57% (6 eyes) at baseline. At week 12, the proportion of grade 3 or 4 had decreased to 9.52% (2 eyes), while the proportion of grade 0 or 1 had increased to 66.67% (14 eyes) (Grade 0–2 vs 3–4, from baseline to week 12; *P* = 0.0063, Fisher’s exact test) (**C**). The Schirmer test revealed aqueous tear production of 10.81 ± 10.60 mm and 12.67 ± 11.40 mm at baseline and week 12, respectively (*P* > 0.05, paired t-test) (**D**). BCVA: Best-corrected visual acuity, logMAR: log of minimal angle of resolution. **P < 0.001, paired t-test.

At baseline, uncorrected visual acuity (UCVA) was 0.95 ± 0.53 log of the minimal angle of resolution (logMAR) (range 0.30–2.00) and best-corrected visual acuity (BCVA) was 0.77 ± 0.51 logMAR. BCVA was significantly improved to 0.30 ± 0.40 logMAR (range − 0.18–1.00) at 1 week; this improvement was well maintained throughout the whole follow-up period (0.26 ± 0.39 logMAR (range − 0.18–1.00), 0.30 ± 0.40 logMAR (range − 0.08–1.00) and 0.27 ± 0.40 logMAR (range − 0.08–1.00) at 4, 8 and 12 weeks, respectively (compared to baseline; *P* < 0.001 for all weeks, paired t-test)) (Fig. 1A).

Corneal fluorescein staining showed 61.90% (13 eyes) to be grade 3 or 4 at baseline. After 12 weeks of the LK scleral lens wearing, the proportion of corneal fluorescein staining grade 3 or 4 had significantly decreased to 14.29% (3 eyes), and a majority of subjects (71.43%, 15 eyes) were either grade 0 or 1 (*P* = 0.0036, Fisher’s exact test) (Fig. 1B). Additionally, 52.38% (11 eyes) had conjunctival fluorescein staining grade 3 or 4, and 28.57% (6 eyes) had grade 0 or 1 at baseline. After 12 weeks, 9.52% (2 eyes) had conjunctival staining grade 3 or 4, and 66.67% (14 eyes) had grade 0 or 1 (*P* = 0.0063, Fisher’s exact test) (Fig. 1C).

Aqueous tear production measured by the Schirmer test revealed no significant difference between baseline (10.81 ± 10.60 mm) and week 12 (12.67 ± 11.40 mm) (Fig. 1D). The overall average of the LK scleral lens wearing time was 55.26 ± 30.26 (range 14.86–106.58) hours per week.

### Subjective efficacy outcomes

**Figure 2:**
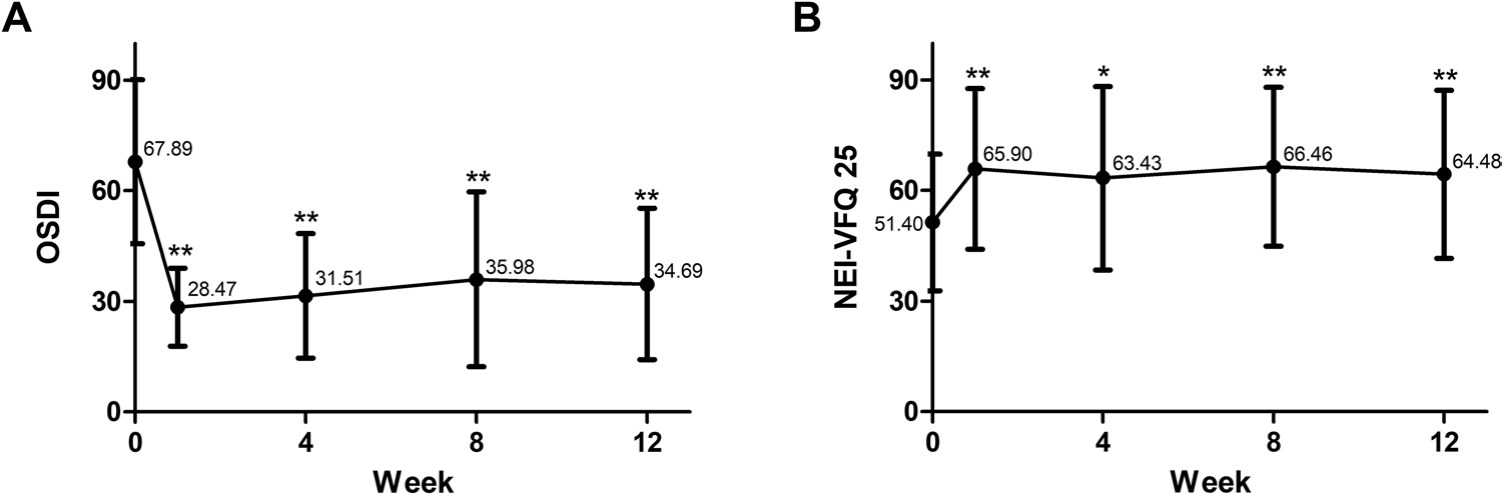
Subjective efficacy outcomes of the LK scleral lens clinical trial. At baseline, OSDI was 67.89 ± 22.29 (range 20.45–100.00), which significantly decreased to 28.47 ± 10.60 (range 6.25–47.73), 31.51 ± 16.88 (range 2.08–60.00), 35.98 ± 23.73 (range 2.50–72.73), and 34.69 ± 20.51 (range 6.82–72.92) at weeks 1, 4, 8, and 12, respectively (compared to baseline; *P* < 0.001 at all weeks) (**A**). NEI-VFQ-25 score was 51.40 ± 18.50 (range 22.00–81.04) at baseline, which significantly improved to 65.90 ± 21.82 (range 22.63–97.05), 63.43 ± 24.89 (range 17.27–97.73), 66.46 ± 21.59 (range 26.50–98.75), and 64.48 ± 22.76 (range 16.59–95.94) at weeks 1, 4, 8, and 12, respectively (compared to baseline; *P* < 0.001 at weeks 1, 8, and 12, *P* < 0.01 at week 4) (**B**). OSDI: Ocular surface disease index, NEI-VFQ-25: National Eye Institute 25-Item Visual Function Questionnaire. *P < 0.01, paired t-test, **P < 0.001, paired t-test.

The ocular surface disease index (OSDI) was 67.89 ± 22.29 (range 20.45–100.00) at baseline and improved to 28.47 ± 10.60 (range 6.25–47.73), 31.51 ± 16.88 (range 2.08–60.00), 35.98 ± 23.73 (range 2.50–72.73), and 34.69 ± 20.51 (range 6.82–72.92) at weeks 1, 4, 8, and 12, respectively (compared to baseline; *P* < 0.001 for all weeks, paired t-test). With the LK scleral lens wearing, OSDI showed a significant decrease of 30 to 40 points after 1 week, which was sustained throughout the study period (Fig. 2A). The National Eye Institute 25-Item Visual Function Questionnaire (NEI-VFQ-25) score was 51.40 ± 18.50 (range 22.00–81.04) at baseline and showed a significant increase to 65.90 ± 21.82 (range 22.63–97.05), 63.43 ± 24.89 (range 17.27–97.73), 66.46 ± 21.59 (range 26.50–98.75), and 64.48 ± 22.76 (range 16.59–95.94) at weeks 1, 4, 8, and 12, respectively (compared to baseline; *P* < 0.001 at weeks 1, 8, and 12, *P* < 0.01 at week 4, paired t-test). NEI-VFQ-25 score increased by more than 10 points from baseline after week 1, which was sustained until the end of the study (Fig. 2B).

### Safety outcomes

**Figure 3:**
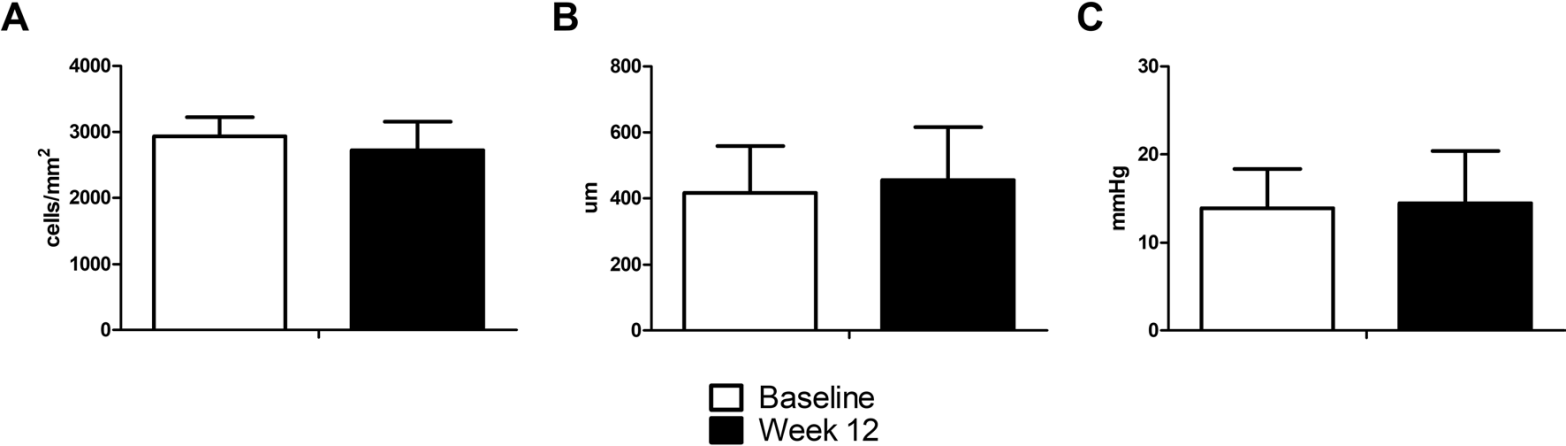
Safety outcomes of the LK scleral lens clinical trial. Endothelial cell density was 2931.4 ± 290.2 cells/mm^2^ at baseline and 2720.1 ± 436.5 cells/mm^2^ at week 12 (*P* > 0.05, paired t-test) (**A**). Central corneal thickness was 416.60 ± 141.54 µm at baseline and 454.70 ± 160.73 µm at week 12 (*P* > 0.05, paired t-test) (**B**). Intraocular pressure was 13.86 ± 4.48 mmHg at baseline and 14.43 ± 5.97 mmHg at week 12 (*P* > 0.05, paired t-test) (**C**).

The specular microscopic data of ten subjects, who had attained clear images at baseline, were used for further analyses. The endothelial cell density (ECD) was an average of 2931.4 ± 290.2 cells/mm^2^ at baseline and 2720.1 ± 436.5 cells/mm^2^ at week 12, respectively (Fig. 3A). The mean coefficient of variation and hexagonality were 36.0 ± 8.0 and 48.1 ± 11.1% at the end of study. Central corneal thickness (CCT) was an average of 416.60 ± 141.54 µm at baseline and 454.70 ± 160.73 µm at week 12 (Fig. 3B). Intraocular pressure (IOP) was an average of 13.86 ± 4.48 mmHg and 14.43 ± 5.97 mmHg at baseline and week 12, respectively (Fig. 3C). None of the above-mentioned safety measurements showed any significant changes, and no sign of infection related to long-term use of the lens was observed.

### Representative case presentation

**Figure 4:**
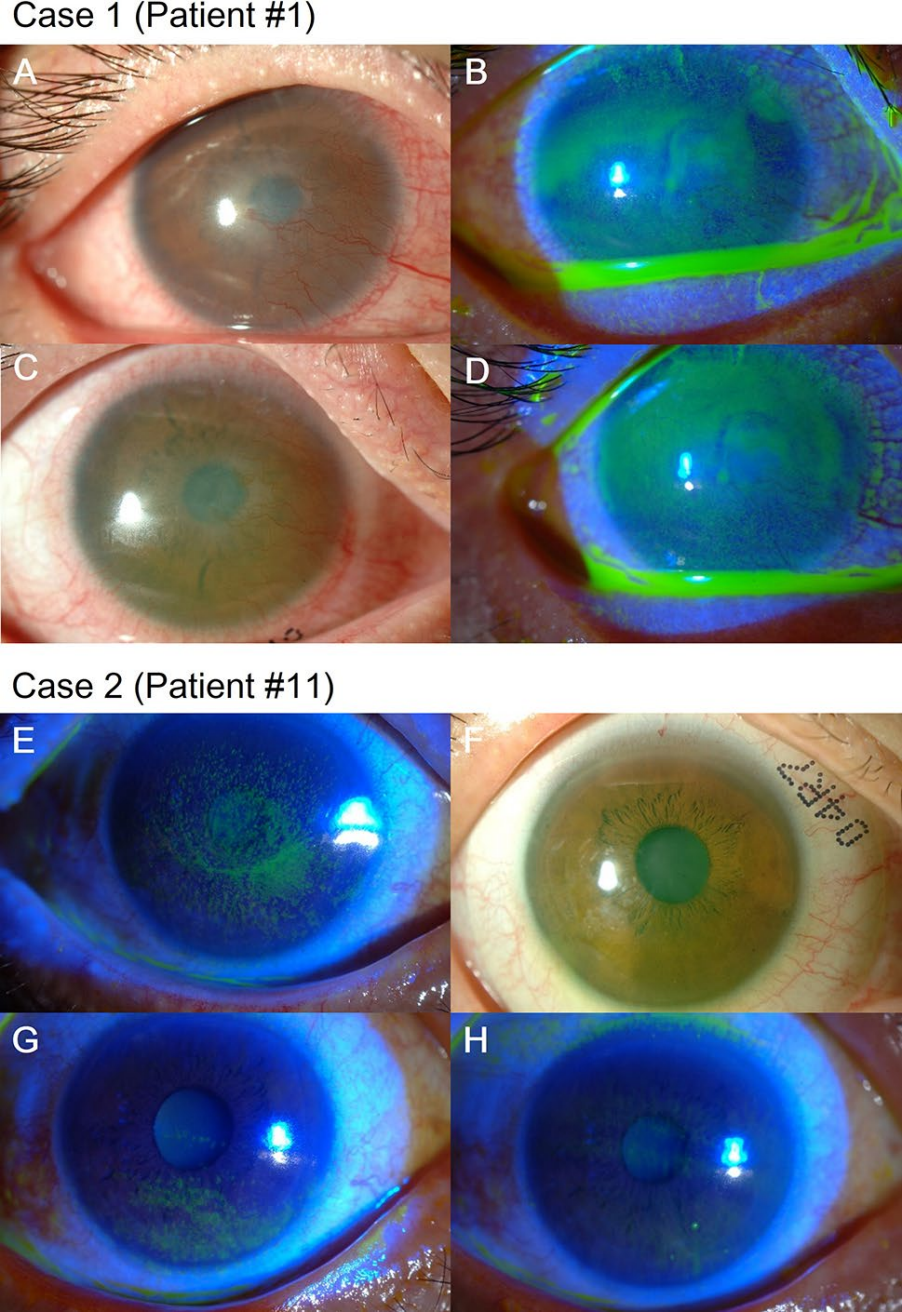
Representative photos of enrolled subjects. Case 1 (Patient #1) shows the right eye of a 23-year-old male with SJS that occurred 6 years ago after taking cold medication. At presentation, both the UCVA and BCVA were 0.82 logMAR. Anterior segment photographs without and with fluorescein application at presentation are shown in (**A**) and (**B**), respectively. LK scleral lens fitting was appropriate with no vascular compression as shown in (**C**). The BCVA after 1 week significantly improved to 0.15 logMAR, which was stably sustained throughout the entire 12-week study period. The corneal fluorescein staining score did not show any significant difference from baseline (**D**). Case 2 (Patient #11) show the left eye of a 53-year-old female with cGVHD that occurred after allogeneic bone marrow transplantation for acute myeloid lymphoma. At presentation, both the UCVA and BCVA were 0.40 logMAR with diffuse corneal punctate epithelial erosions (**E**). LK scleral lens fitting was appropriate with no vascular compression as shown in (**F**). With the LK scleral lens, the erosions had grossly decreased at week 1 (**G**) and continued to improve with only minimal erosions remaining by week 12 (**H**). BCVA was − 0.08 logMAR at week 1, which was maintained until the end of the study. BCVA: Best-corrected visual acuity, cGVHD: chronic graft-versus-host disease, logMAR: log of minimal angle of resolution, SJS: Stevens–Johnson syndrome, UCVA: uncorrected visual acuity.

Representative anterior segment photos are shown in Fig. 4. Case 1 (Patient #1) shows the right eye of a 23-year-old male with SJS that occurred 6 years ago after taking cold medications. At presentation, both UCVA and BCVA were 0.82 logMAR. Slit-lamp examination showed central corneal opacity with neovascularization and severe corneal and conjunctival fluorescein staining (Fig. 4A,B). The LK scleral lens fitting was appropriate without vascular compression (Fig. 4C). The BCVA after 1 week dramatically improved to 0.15 logMAR, which was sustained throughout the entire study period. Corneal fluorescein staining score did not show any significant difference (Fig. 4D). Case 2 (Patient #11) shows the left eye of a 53-year-old female with cGVHD that occurred after allogeneic bone marrow transplantation for acute myeloid lymphoma. At presentation, both UCVA and BCVA were 0.40 logMAR with diffuse corneal punctate epithelial erosions (Fig. 4E). The LK scleral lens fitting was appropriate with no vascular compression (Fig. 4F). With the LK scleral lens, corneal punctate epithelial erosions had grossly decreased at week 1 (Fig. 4G) and continued to improve with only minimal erosions by week 12 (Fig. 4H). BCVA was − 0.08 logMAR at week 1, which was maintained until the end of the study.

## Discussion

In our study, we observed the beneficial effects of the LK scleral lens with large OAD for various intractable ocular surface diseases, such as PED, cGVHD, SJS and severe dry eye. BCVA significantly improved from an average of 0.77 ± 0.51 logMAR at baseline to 0.27 ± 0.40 logMAR with the LK scleral lens use at the end of this 12-week study. This vision-improving effect was evident from week 1. The proportion of low-grade fluorescein staining grades of both cornea and conjunctiva significantly increased at the end of the study. Subjective dry eye symptoms were significantly ameliorated by week 1 of the LK scleral lens use, which was stably maintained throughout the entire study period. Additionally, we did not find any clinically significant complications.

Over the years, many studies have proven the efficacy of scleral lenses in managing various ocular surface diseases. Scleral lenses have now become a primary or secondary treatment of choice for intractable ocular surface diseases^18^. Due to the full cornea-covering and rigid design of the scleral lens, the tear reservoir between the ocular surface and the scleral lens provides prolonged tear lubrication to improve dry eye and help even out the ocular surface subsequently reducing optical aberrations and improving vision^2,13,16,19^. Long-term use of PROSE has been reported to improve visual acuity by 0.20–0.46 logMAR and to successfully sustain vision for several ocular surface diseases and irregular corneas^20^. Heur et al. reported the PROSE treatment to be a viable option for SJS patients when conventional treatments have failed^7^. Similarly, Papakostas et al. also found the PROSE treatment to offer significant improvement in visual function and acuity in SJS patients^15^. Jacobs and Rosenthal observed that Boston scleral lenses help enhance the quality of life of patients with cGVHD^9^. Schornack reported that Jupiter scleral lenses help buy time for some patients with limbal stem cell deficiency until aggressive surgical measures are inevitable^21^. Despite the variety of commercially available scleral lenses today, most studies have focused on using PROSE and Jupiter Lens^5–7,20–24^. There have been only a few studies regarding other scleral lenses^4,6,8,25^. Recently, the SoClear lens (Art Optical Contact Lens, Inc., Grand Rapids, MI, USA), a commercially available corneoscleral lens with a diameter of 14.0 mm, was reported to have long-term efficacious results in improving corneal fluorescein staining and visual acuity in patients with refractive ocular surface diseases, such as SJS and cGVHD^14^. Similar to past studies mentioned above, our study also demonstrated clinical benefits and comparable efficacy of the LK scleral lenses for various intractable ocular surface diseases, including PED, cGVHD, SJS and severe dry eye. In this study, the LK scleral lens enhanced visual acuity by approximately 0.47 logMAR at week 1, which was sustained throughout the 12-week study period. The proportion of subjects with high-grade corneal and conjunctival fluorescein staining had significantly decreased by week 12. Subjective dry eye indices significantly improved by nearly 40 points in the OSDI and 15 points in the NEI-VFQ-25 score.

Meanwhile, we found that, compared to female subjects, final BCVA of male participants improved significantly by − 0.65 ± 0.34 logMAR from baseline BCVA (*P* = 0.005, unpaired t-test). However, there was no difference depending on age or disease status. In addition, we were able to observe high compliance with the LK scleral lens wearing, in which all of our subjects wore the LK scleral lens for an average of 55 h per week. Among them, the LK scleral lens were worn on 8 eyes for an average of 12 h or more daily. Compared to the participants who wore the LK scleral lens less than 12 h daily, they had better BCVA at the end of study (0.11 ± 0.18 vs. 0.50 ± 0.39, *P* = 0.031, Wilcoxon rank sum test). There were 16 eyes subjected to overnight LK scleral lens wear, and these were observed to have significantly reduced limbal redness, corneal and conjunctival fluorescein staining, and corneal ulcer at the end of the study compared to those that did not (all *P* < 0.05, Cochran–Mantel–Haenszel test). Especially among those who had persistent epithelial defects and had worn the LK scleral lenses overnight, many experienced alleviations of epithelial defects.

Most scleral lenses are manufactured in the United States of America, England, the Netherlands, and Israel, which may limit import or accessibility in some countries. Moreover, since their manufacturers are mainly in the western world, these scleral lenses may not fit Asian subjects perfectly. The LK scleral lenses used in this study are distinct from other scleral lenses, in that they are custom-made to fit Asian eyes, which possess smaller palpebral fissures and tighter eyelids than the eyes of Western populations. Most of our study subjects wore the LK scleral lenses designed with an OAD of 17.5–18.5 mm, which are relatively smaller than the conventionally used scleral lenses in the Western population and larger than the commercially available corneoscleral lenses in the Republic of Korea. Despite the larger OAD of the LK scleral lens compared to that of commercially available scleral lenses in the Republic of Korea, we observed that only one subject had difficulty in handling the LK scleral lens among the initial 16 subjects enrolled. With intensive education regarding the insertion and removal of the LK scleral lens, it was fully possible for Koreans to successfully use the LK scleral lens. Of the 13 subjects that were ultimately enrolled in our study, the average number of trial lens fittings and visits for fitting were 4.33 lenses and 2.24 times, which were similar to previous studies reporting 1.5–3.4 lenses and 2.8–5.86 times, respectively^26–29^.

Although many studies have reported the safety of scleral lenses, corneal edema or endothelial dysfunction remain among the complications of and contraindications to scleral lens treatment^23,30^. In a study by Schear et al., scleral lens treatment failure was observed in 6.4% of patients, and the main reason for failure was worsening of corneal edema^23^. It was reported that diseases with presumed risk factors for low ECD were more likely to fail scleral lens treatment^23^. In this study, there were no statistically significant changes in ECD and CCT induced by wearing the LK scleral lens, although visualization of endothelial cells was limited in some patients and the value of CCT might be incorrect due to hazy and irregular nature of the ocular surface. Still, the possibility of endothelial decompensation should be monitored during scleral lens use in all subjects, especially for those who already possess low ECDs. Considering that this was a 12-week study, which is an inadequate amount of time to precisely determine the effect of the LK scleral lens on the endothelium, a future study with a longer period of the LK scleral lens use is warranted. Meanwhile, in 3 participants, clearer images of endothelial cells were observed at the end of study compared to those at initial screening. This suggests that the LK scleral lens can help improve corneal edema, haziness or irregularity, and facilitate more precise ECD measurements. Regarding disease-specific findings and other common contact lens-related complications, we did not observe any change in blepharitis, conjunctivitis, endothelial blebs, corneal distortions, corneal neovascularization or epithelial microcysts after 12 weeks of the LK scleral lens use (all *P* > 0.05, Bowker’s test).

There are some limitations in this study. First, we could not conduct clinical trials using scleral lenses with smaller OAD as a control. Nevertheless, we carefully think that the efficacy of the LK scleral lens is not less than that of scleral lenses with smaller OAD. Although inclusion criteria and study population are a little bit different, a previous study using SoClear in Korean subjects with severe ocular surface diseases would be a good comparative reference^14^, which demonstrated that visual acuity and corneal fluorescein staining significantly improved, while there was no significant difference in subjective dry eye symptoms. On the other hand, in the current study, the LK scleral lenses showed significant improvement not only in vision and fluorescein staining but also in subjective dry eye symptoms. This may be attributed to the larger OAD of 17.5–18.5 mm of the LK scleral lens that enables it to retain a better tear reservoir compared to SoClear which possesses a smaller OAD of 14.0 mm. The relative small sample size is one of the limitations of this study. Considering that the LK scleral lens has not been approved as a medical device in the Republic of Korea, the recruitment of subjects with visual acuities better than 0.3 logMAR was not ethically possible. Based on the current study, the LK scleral lens has obtained the approval for grade 3 medical devices, and an expanded application to subjects with better vision is now possible. Therefore, future studies with a larger number of subjects using the LK scleral lenses may provide better information. Additionally, our study period was only 12 weeks, which is not a sufficient length of time to determine the long-term beneficial and adverse effects of the LK scleral lens. The heterogeneity of our study subjects is another limitation. Further studies with the LK scleral lenses with a more homogenous study group may better highlight the specific benefits of the LK scleral lenses.

In conclusion, the LK scleral lenses successfully provided subjects suffering from intractable ocular surface diseases with alleviation of both objective signs and subjective symptoms of their diseases and improved visual acuity without any clinically significant complications.

## Methods

This prospective study adhered to the ethical standards of the Declaration of Helsinki and was approved by the Institutional Review Boards of Seoul National University Hospital (IRB number: D-1008-107-328) and Seoul National University Bundang Hospital (IRB number: E-1009/054-001). Written informed consent was obtained from all participants.

### The LK scleral lens

The LK scleral lens is a concentric symmetrical lens employing spherical landing zones without fenestrations. The central thickness of the lenses ranges from 0.22 to 1.48 mm. The LK scleral lens possesses five curves configured into three zones: (1) the central (optic) zone, with an asphericity of 0.5 and that lies above the cornea, (2) the transitional zone (2 curves), which joins the central and landing zones and vaults the limbus and (3) the landing zone (2 curves). The sagittal height is controlled by each curvature of the transitional zone, which can be lengthened or shortened in 0.05-mm steps.

### Study design

This was a prospective, open-label and single-arm clinical trial performed at two tertiary referral hospitals to ascertain the clinical effects and safety of the LK scleral lens in patients with intractable ocular surface diseases.

Clinical trials were performed individually at Seoul National University Hospital (Seoul, Republic of Korea) and Seoul National University Bundang Hospital (Seongnam-si, Gyeonggi-do, Republic of Korea) between May 1, 2012, and March 31, 2014. Subjects were enrolled according to the inclusion and exclusion criteria shown in Table 2. Briefly, this study included subjects between 20 to 69 years of age with intractable ocular surface diseases such as severe dry eye (dry eye symptoms with Schirmer test ≤ 5 mm), PED, chemical burn, SJS, ocular pemphigoid or cGVHD were enrolled. It was considered intractable when despite, extended use for more than 6 months of medical treatments such as artificial tears, topical steroids and autologous serum eyedrops, clinical responses were insufficient and minute. Additionally, patients with an UCVA better than counting finger and worse than 0.3 logMAR with a BCVA worse than 0.3 logMAR were included.

**Table 2:** Inclusion and exclusion criteria.

Inclusion
A. Age between 20 to 69 years
B. Intractable ocular surface diseaseª
C. UCVA > counting finger and ≥ 0.3 logMAR
D. BCVA ≥ 0.3 logMAR

Exclusion
A. Currently under treatment for infectious keratitis
B. Difficulty of scleral lens application due to severe allergic conjunctivitis, low compliance, and other potential contraindications
C. Diabetes mellitus and/or hypertension
D. Pregnant or planning pregnancy
E. Unwilling to follow study schedule
F. Enrolled in another clinical study
G. Determined inappropriate under investigators’ discretion

ªDiagnosed with either of the following intractable ocular surface disease; severe dry eye (dry eye symptoms with Schirmer test ≤ 5 mm), persistent epithelial defect, chemical burn, Stevens–Johnson syndrome, ocular pemphigoid or chronic graft-versus-host disease. Also, clinically refractive to medical treatment, such as artificial tears, topical steroids and autologous serum eyedrops, for more than 6 months.

UCVA: uncorrected visual acuity, logMAR: log of minimal angle of resolution, BCVA: best-corrected visual acuity.

### Screening and trial scleral lens fitting

**Figure 5:**
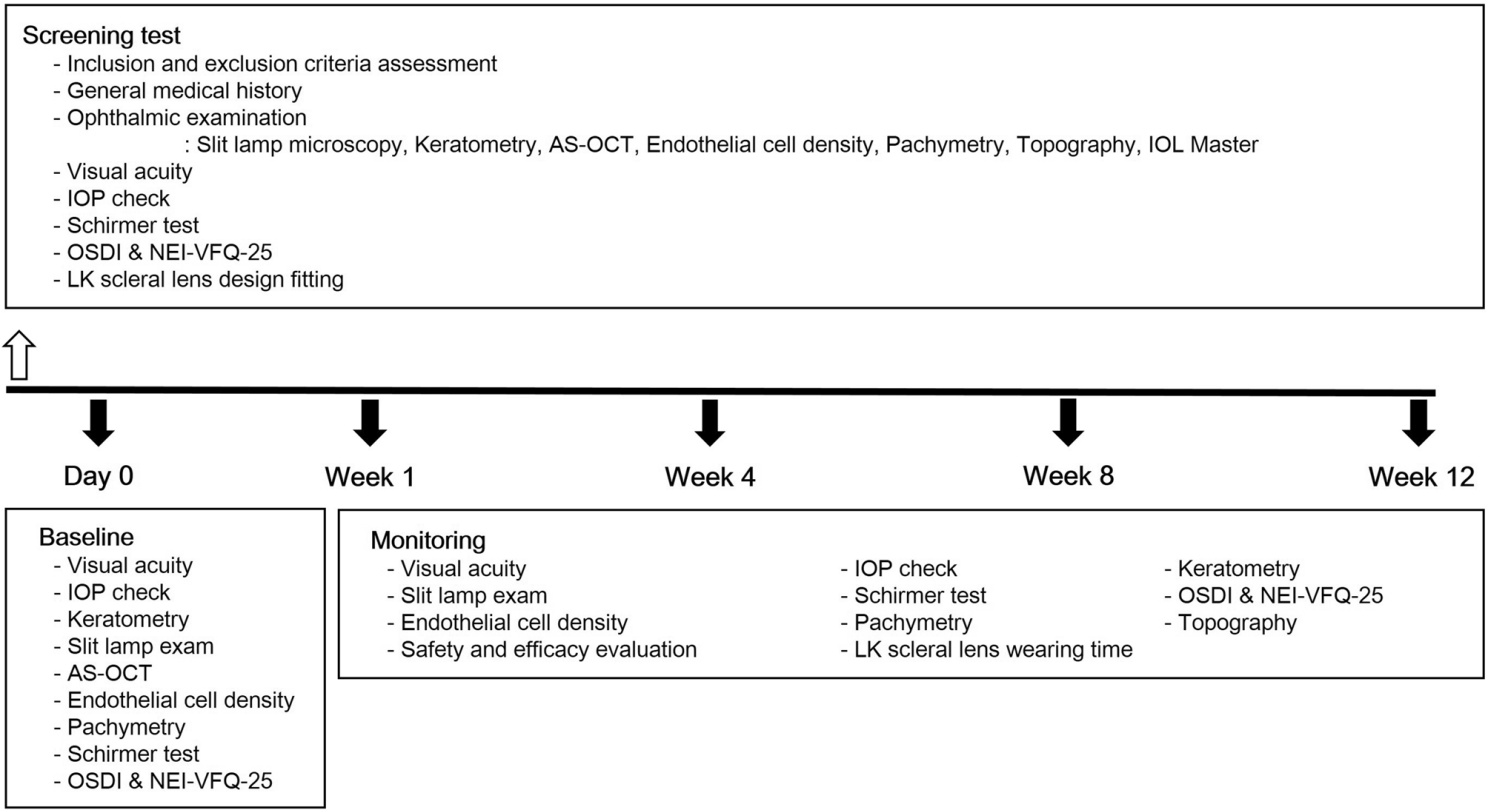
Flow chart of the study schedule. All subjects underwent basic screening tests. When determined applicable, individual LK scleral lens fitting was performed. Subjects visited the clinic at day 0, weeks 1, 4, 8 and 12. At every visit, all subjects received thorough ophthalmic examinations with questionnaires to fully evaluate the wearing time, compliance, and safety and efficacy of the LK scleral lens. AS-OCT: anterior segment optical coherence tomography, IOP: intraocular pressure, OSDI: ocular surface disease index, NEI-VFQ-25: National Eye Institute 25-Item Visual Function Questionnaire.

At the screening visit, general demographics and medical history were collected from all subjects. All subjects underwent the basic screening tests listed in Fig. 5. When the participant’s conditions met the inclusion and exclusion criteria and were determined applicable, the participant subsequently proceeded to individual trial LK scleral lens fitting.

**Figure 6:**
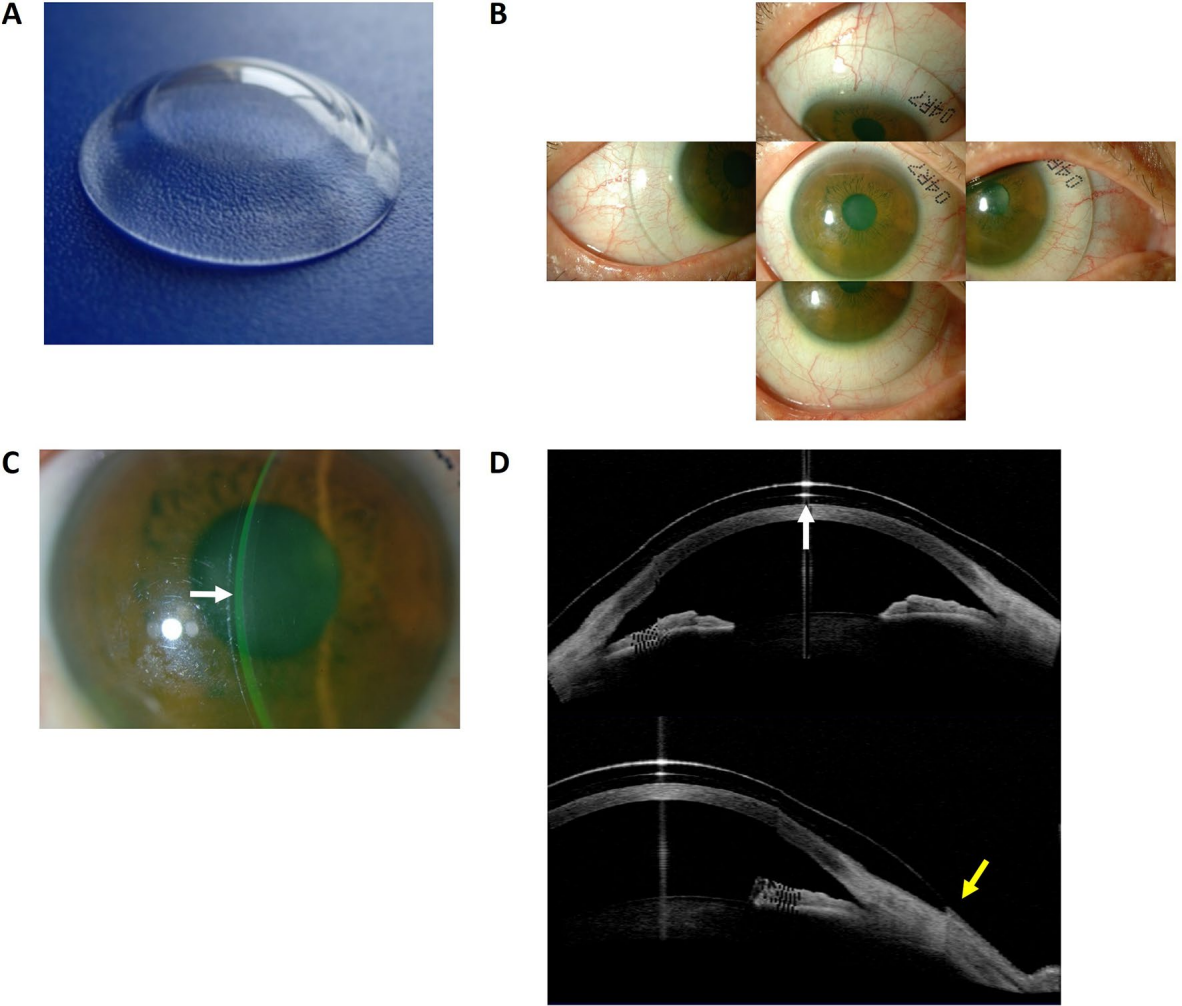
Individual LK scleral lens design and custom fitting. The LK scleral lens was custom-made (**A**) to fit each subject individually based on different ophthalmic examinations, including corneal topography and anterior segment optical coherence tomography. Absence of vascular compression at all gazes including primary position was confirmed under slit-lamp biomicroscopy examination (**B**). A tear-filled vault over the cornea of approximately half of the central corneal diameter was considered appropriate (**C**). Sagittal depth and overall LK scleral lens fitting were also assessed using anterior segment optical coherence tomography (**D**). White arrow indicates ideal tear reservoir over the cornea. Yellow arrow indicates absence of conjunctival vascular compression.

The LK scleral lens fitting was performed in a customized manner. The OAD was determined according to the horizontal visible iris diameter, the width and height of the eyelid fissure, and the eyelid tension. The optic zone diameter was 9.0 mm, and the base curve radius was selected based on the subject’s topographic findings (Orbscan II, Bausch & Lomb, Rochester, NY, USA). The landing zone curvatures were determined by estimating the scleral curvature using anterior segment optical coherence tomography (AS-OCT, Visante OCT, Carl Zeiss Meditec AG, Jena, Germany) as described in a previous study^31^. The AS-OCT was also used to estimate the sagittal height of the fitted area, by which the curvatures of the transitional zone were decided. Scleral lens fitting was initially considered correct when clearance of fluorescein was seen under slit-lamp biomicroscopy and the tear-filled vault over the central cornea was more than half of the CCT without limbal or scleral vessel compression. Appropriate fitting was confirmed by adjusting the above mentioned parameters based on the findings of slit-lamp biomicroscopy and AS-OCT (Fig. 6). After the initial fitting, the subject was examined for 2 h to determine the settling back effect. The power of the lens was determined by spherical overrefraction using an autorefractometer (KR-8100, Topcon, Tokyo, Japan). In case overrefraction was not possible due to corneal haziness, a spherical lens based on the parameters of each participant’s previous glasses was tried empirically. After delivery of the ordered lens, the lens fitting status was checked again for at least 2 h, and the lens was finally dispensed to each participant (Day 0, baseline). All scleral lenses were cleansed with standard RGP cleaning solutions and was filled with normal saline for wearing.

### Follow-up schedule and monitoring

The follow-up schedule and monitoring are shown in Fig. 5. Participants were advised to wear the scleral lens at least 30 h per week and visit the clinic at weeks 1, 4, 8 and 12 after the LK scleral lens wearing. At every visit, all subjects received thorough ophthalmic examinations with questionnaires to fully evaluate the wearing time, compliance, and safety and efficacy of the LK scleral lens. During the study period, all subjects maintained their current therapies such as artificial tear eyedrops or ointments, topical corticosteroids, topical cyclosporines or autologous serum eyedrops.

Primary outcome measures were the efficacy and safety of the LK scleral lenses. Objective efficacy was evaluated with visual acuities, where Snellen values were converted to logMAR scales, corneal and conjunctival staining and aqueous tear production using the Schirmer test^32^. Corneal and conjunctival fluorescein staining were evaluated using the Oxford grading system^33^. Subjective efficacy was evaluated with the OSDI^34^ and NEI-VFQ-25^35,36^. OSDI is an efficient dry eye questionnaire to evaluate both frequency of dry eye symptoms and their current influence on visual function, and defines dry eye when the score is 13 or more^37^. NEI-VFQ-25 is a questionnaire beneficial in assessing the overall dry eye symptoms’ impact on quality of life where scores can range from 0 to 100 (lower scores indicate more problems or symptoms)^38,39^. Safety was assessed with ECD using a noncontact specular microscope (Konan Medical Inc., Hyogo, Japan), CCT using ultrasound pachymetry (Quantel Medical, Clermont-Ferrand, France), and IOP with a noncontact tonometer (CT-800A, Topcon, Tokyo, Japan) and inspection for clinical signs of infection related to long-term scleral lens wearing.

### Statistical analysis

Statistical analysis was performed using SPSS software for Windows version 22.0 (SPSS, Inc., Chicago, IL). The Wilcoxon signed-rank test, 2-tailed Fisher’s exact test and paired t-test performed at the 95% confidence interval were used to assess outcome measurements before and after scleral lens use. Unpaired t-test, Bowker’s test, and Cochran–Mantel–Haenszel test were used for subgroup analysis. A probability value of < 0.05 was considered statistically significant. The results are presented as the means ± standard deviations (SDs) unless otherwise indicated.

### Ethics approval and consent to participate

This prospective study adhered to the ethical standards of the Declaration of Helsinki and was approved by the Institutional Review Boards of Seoul National University Hospital (IRB number: D-1008-107-328) and Seoul National University Bundang Hospital (IRB number: E-1009/054-001). Written informed consent was obtained from all participants.

### Consent for publication

Written informed consent was obtained from all participants.

## Data Availability

All data generated or analysed during this study are included in this published article.

## Abbreviations

AS-OCT: Anterior segment optical coherence tomography
BCVA: Best-corrected visual acuity
CCT: Central corneal thickness
cGVHD: Chronic graft-versus-host disease
ECD: Endothelial cell density
IOP: Intraocular pressure
logMAR: Log of the minimal angle of resolution
NEI-VFQ-25: National Eye Institute 25-Item Visual Function Questionnaire
OSDI: Ocular surface disease index
PED: Persistent epithelial defect
PROSE: Prosthetic replacement of the ocular surface ecosystem
SD: Standard deviations
SJS: Stevens–Johnson syndrome
UCVA: Uncorrected visual acuity
